# Instability of emotional relationships and suicide among youth: a qualitative study

**DOI:** 10.1186/s12888-023-04534-0

**Published:** 2023-01-18

**Authors:** Masoud Fallahi-Khoshknab, Zainab Amirian, Sadat Seyed Bagher Maddah, Hamid Reza Khankeh, Asghar Dalvandi

**Affiliations:** 1 Nursing Department, University of Social Welfare and Rehabilitation Sciences (USWR), Tehran, Iran; 2 Nursing Department, Health in Emergencies and Disasters Research Center, University of Social Welfare and Rehabilitation Science (USWR), Tehran, Iran; 3 Department of Clinical Science and Education, Karolinska Institute, Stockholm, Sweden

**Keywords:** Suicide, Poisoning, Qualitative study, Emotional stress

## Abstract

**Background:**

Interpersonal problems are one of the factors for understanding the complex issues that result in suicide attempts and self-injury by poisoning. The quality of familial relationships is a predictor of the occurrence and outcome of suicide attempts. This study aimed to explore motives for self-poisoning suicide attempts amongst young adults.

**Method:**

This research was a qualitative study conducted using semi-structured interviews in 2019 in Kermanshah Province, Iran. Eighteen participants who had attempted suicide by self-poisoning were interviewed, and information was collected until data saturation was achieved. The interviews were recorded and transcribed, and the data were analyzed through content analysis.

**Results:**

The results included the category of instability in emotional relationships with the three sub-categories of 1- Emotional failure, 2- Emotional trauma, and 3- Loss of emotional resilience (caused by emotional failure and emotional trauma within the previous few months). Instability in emotional relationships creates feelings of disgrace, humiliation, burdensomeness, worthlessness, and insignificance, which increases the chances of attempting suicide.

**Conclusion:**

The study results provided an in-depth understanding of romantic, and unstable familial relationships as a significant factor in suicide attempts, demonstrating the role of emotional stress in attempting suicide. The present study provided information on the risk factors and warning signs for psychiatrists and nurses dealing with suicidal patients to take effective measures to prevent suicide through social support.

## Introduction

Suicide, defined as an intentional self-inflicted death, remains a global public health issue, accounting for around 800 thousand deaths per year worldwide [1]. One of the oldest ways of attempting suicide is self-poisoning [2]. Suicide through self-poisoning occurs most commonly using medication, chemical agents (pesticides), poisons, or illicit drugs [3]. In Iran, self-poisoning suicide is more prevalent among people aged between 20 and 30 [4].

Self-poisoning is a common cause of hospitalization. It is associated with complex and serious health problems and poses complex issues for health professionals in primary and secondary care [5]. Suicide among youth happens due to a wide range of complex causes [6]. Increase in stress and pressure in life, a decrease in mental strength to deal with stress, easy access to methods of suicide, social issues such as marital disputes, economic problems, reproach, unemployment, and quarrels are factors contributing to suicide [7] Mueller et al. (2015) reported that both boys and girls are more likely to report emotional stress and suicidal ideation after the suicide attempt of a friend [8]. In a comprehensive review study on Muslim women, Canetto et al. (2015) posited that relationship problems can take a variety of forms [9]. A change in communication distance (reduction in closeness or intimacy) is predictive of the nature and severity of suicide attempt and its consequences [10]. Those who attempt suicide in front of a spouse or lover are often reacting to some reduction in closeness – what Black calls “under-intimacy.” Under-intimacy includes separation, divorce, and infidelity, all of which cause severe conflict between intimate partners [10, 11].

In Joiner’s interpersonal theory of suicide (IPTS), one of the constituents of suicidal desire is a thwarted sense of belonging. Joiner’s (2005) view is that “the need to belong is so powerful that, when satisfied, it can prevent suicide even when a feeling of burdensomeness and the ability to enact lethal self-injury are in place” [12]. An ethnographic psychologist, Jeanne Marecek (2006), reported that interpersonal problems are among the factors for understanding the complex issues that result in a suicide attempt and self-injury by poisoning, which occur in the context of family disputes and other conflicts with intimate people in relation to social and ecological conditions [13]. Youths who feel that they have close relationships with their peers, parents, and family members and youths who feel they are part of their school or society are less likely to have serious thoughts about suicide than their counterparts [14, 15]. Paths that lead to attempting suicide using different methods, such as self-poisoning, are complex, and conducting studies on suicide attempts can lead to a more extensive understanding of this phenomenon and help prevent it [16]. According to the World Health Organization (WHO) [17], 78% of deaths resulting from suicide occur in low-income countries, including many Muslim nations. A study conducted by Eskin et al. [18] called for in-depth studies on suicidal behaviors, especially in Muslim-majority countries. The results of a systematic review in Iran revealed that family and familial disputes are the cause of 26–30% of suicides [19].

People often attempt suicide after an adverse event, and the most common events experienced before suicide are social, familial, and marital problems [20]. Moreover, the characteristics of families are different in different cultures and nations [21]. In a meta-analysis of suicide risk factors in the last 50 years, Franklin et al. [22] reported that these risk factors have limited value in understanding and preventing suicide. Considering the high prevalence of suicide among youth, the limited value of risk factors in understanding it, its complexity and multidimensionality, its dependence on socioeconomic conditions, its interpersonal nature, and the differences between families in different nations, qualitative research on this issue will help us achieve a more holistic understanding of this phenomenon. Iran is a predominantly Muslim country where suicide is forbidden in the religious and national culture and there is great emphasis on familial support. Qualitative approaches are helpful for in-depth studies on unknown phenomena with socio-cultural aspects. They have the potential to discover the symbolic meaning of social phenomena [23, 24]. Suicidology cannot be counted as an independent scientific discipline but rather as a multidisciplinary area with inherent problems such as translation between different cultures and the consequent difficulties in forming coherent models and theories. Consequently, it is necessary to focus on specific aspects of suicidal behaviour instead of working on general models [25]. “We believe that suicide, as a social and interpersonal phenomenon in Iran, has not been sufficiently analyzed”. Therefore, conducting qualitative research on relational problems and emotional conflicts can provide a more comprehensive understanding of suicide prevention. This study aimed to explore motives for suicide attempts by self-poisoning amongst young adults.

## Methods

### The present study

This study was a qualitative study conducted using conventional content analysis. This method directly extracts information from participants without any prior hypothesis. The information is generated based on participants’ unique views. Codes, sub-categories, and categories are derived using the inductive approach, arranged as concepts, and developed based on their characteristics and dimensions. Themes or hidden and visible patterns are revealed in data. In conventional content analysis, researchers avoid using predetermined categories and allow the categories to emerge from the data [26].

### Study setting

The study was conducted in Kermanshah, a province in the west of Iran. The main admission center for poisoned patients in Kermanshah is Imam Khomeini Hospital, with 220 beds; this hospital is affiliated with Kermanshah University of Medical Sciences and is the referral center for poisoning cases in the west of the country. The young people who were hospitalized in the emergency/poisoning department of Imam Khomeini Hospital due to suicide attempts by self-poisoning were 19–31 years old, had overcome the acute phase, and were conscious and willing to share their experiences were included in the study. The interviews were conducted in private rooms or at the bedside of patients.

### Data collection

Participants were selected by purposive sampling among patients who had attempted suicide by self-poisoning with maximum diversity in age, gender, marital status, and education. Sampling continued until data saturation was reached and no new category or sub-category was obtained. Data collection and analysis were done simultaneously. Semi-structured, in-depth, individual, face-to-face interviews were conducted for data collection. The interviews took 30–45 minutes and were conducted at the time and place in the emergency/poisoning department of Imam Khomeini Hospital that was convenient for the participants, either at the patients’ bedside or in private rooms; eighteen interviews were conducted. The main questions included, “Why were you hospitalized?”, “Please tell us what made you attempt suicide,” or “Please tell the story of the day you attempted suicide.” Afterward, follow-up questions determined by the primary answers were asked for clarification.

### Data analysis

Data analysis was done according to Graneheim and Lundman’s approach [26]. The interviews were recorded and transcribed as soon as possible after the interview. The transcripts were reviewed several times to achieve a general understanding; then, they were divided into meaning units, and the meaning units were summarized and coded. Afterward, different codes were compared and placed in categories and sub-categories based on similarities, differences, and content homogeneity. The initial transcripts and final codes were reread several times until the researcher and participants reached a shared understanding regarding the concepts. Moreover, the researcher tried to avoid their presumptions as much as possible. All the coding stages were done using the MAXQDA software.

### Rigor

Four criteria of credibility, dependability, confirmability, and transferability were used to check the trustworthiness of the data. Sufficient time was dedicated to collecting data, conducting interviews, and doing peer checks and member checks to ensure the credibility of the data. Supervisors and advisors checked the transcripts. We tried to explain all stages, including data collection, data analysis, and procedures, so that others could evaluate the study results by reading them to ensure confirmability. Member check was done by summarizing the analyzed interviews and returning them to the participants to know whether the categories that emerged from it were correct to ensure the dependability of the data. For peer check, supervisors and advisors thoroughly discussed the emerged data throughout the research process. Moreover, the study limitations, data collection and analysis, participant selection, and subject description were clearly stated to improve study transferability, so other researchers may continue the study.

### Protection of human participants

An ethics code (IR.USWR.REC.1397.079) was acquired from the university Ethics Committee. Other measures taken included ensuring participants’ awareness of research objectives, acquiring their permission to record the interviews, and assuring them of the confidentiality of the recorded information and their right to withdraw at any time.

## Results

Eighteen patients who had attempted suicide by self-poisoning participated in this study; they included ten women and eight men aged 19–31; ten participants were single, six were married, one was a widow, and one was a divorcee (Table 1). The concept of instability in emotional relationships was extracted from the participants’ deep descriptions (Table 2).

**Table 1:** Participants’ demographic characteristics

Number	Age	Gender	Marital status	Education level	Occupation
1	31	Female	Married	High school diploma	Homemaker
2	23	Female	Single	3rd grade high school	Homemaker
3	21	Female	Single	MA in psychology	Unemployed
4	28	Female	Widow	3rd grade middle school	Homemaker
5	20	Female	Single	High school diploma	Unemployed
6	20	Female	Single	High school diploma	Homemaker
7	21	Male	Single	2nd grade secondary school	Soldier
8	24	Male	Single	High school diploma	Soldier
9	23	Male	Married	High school diploma	Unemployed
10	24	Female	Divorcee	High school diploma	Homemaker
11	20	Male	Single	1st grade secondary school	Unemployed
12	26	Male	Single	Veterinary PhD	Student
13	31	Male	Married	High school diploma	Unemployed
14	30	Female	Married	Law PhD candidate	Clerk
15	28	Male	Married	High school diploma	Unemployed
16	25	Female	Single	High school diploma	Day laborer in an automobile manufacturing company
17	30	Female	Married	High school diploma	Homemaker
18	19	Male	Single	High school diploma	Student

**Table 2:** Categories, sub-categories, and codes

Category	Sub-category	Codes
**Instability in emotional relationships**	Emotional failure)unfaithfulness/cheating in relationships(	-Betrayal of the spouse - Failure in a romantic relationship
	Emotional trauma	- Lack of familial support (rejection by family) - Death of relatives - Sexual assault
	Loss of emotional resilience	- Vicious cycle of problematic emotional events - Familial crisis resulting from divorce - Disruption in emotional relationships (love life and family) - Familial crisis resulting from disability

## The category of instability in emotional relationships

Instability in emotional relationships refers to disruptions of interpersonal relationships between people who have intimate relationships and care about these relationships. Separation from these people can be stressful and lead to suicide. This category includes sub-categories of emotional failure (instability in family), emotional trauma, and loss of emotional resilience, each explained with relevant quotations below.

### Emotional failure (unfaithfulness/cheating in relationships)

One sub-category of instability in emotional relationships is emotional failure, which refers to damage to interpersonal relationships (intimate and close relationships) that are important to people; This disruption can cause conflicts and interpersonal quarrels, and the resulting feelings of disgrace and worthlessness led to suicide attempts in this sample group. This sub-category includes the items mentioned below.

#### Betrayal of the spouse

When one of the spouses realizes that the other has betrayed them and has been involved in extramarital relationships with one or more individuals through checking their partner’s messages or based on the other’s absence at night, they feel betrayed and sometimes worthless – feelings that are sometimes accompanied by fear of public disgrace. In some cases, one imposes limitations on the other’s social interactions to prevent further betrayal. Sometimes, one accuses their spouse of betrayal, and the other feels worthless and attempts suicide to prove their innocence. Limiting social interactions, accusing the spouse of betrayal, and feeling betrayed are associated with cold relationships, interpersonal conflicts, and even physical struggles between spouses. These can lead to suicide attempts in both spouses, especially in men who consider their wife’s betrayal as a sign of dishonor, either threatening their spouse with death or feeling disgraced and attempting suicide themselves.

*“I made a mistake, and this is my life now; I lost everything. A boy gave me his number, and I chatted with him for a while; My husband found out and recorded my voice and threatened to tell my family; I poisoned myself because I knew my family would kill me. Now, I am rejected by my husband and my family. My husband says you have betrayed me; we followed you and saw that you went to his house willingly to have sex.”* (P 1).

In some cases, an individual accuses their spouse of betrayal and tries to limit their social interactions, leading to physical conflicts. Quarrels result when these limitations are broken. When someone cannot change the circumstances and the accusations worsen if they argue, they feel worthless and disgraced, which can lead to suicide attempts.

*“My husband beats me. He is insane. He was only pretending to be cool the first six months. I went out to buy some books yesterday, and he asked me why I had bought the books. He beat me again two nights ago. When I came home from my classes, he asked what happened in the class. I said the professor wrote his phone number on the board, and he said I had a boyfriend and I called him the professor to fool him. He has made me use a simple phone so that I cannot use WhatsApp and Telegram. He accuses me so often that I hate myself now. The only reason I have not complained is that I wanted to protect my marriage; I thought he might come to his senses, but it’s no use, and he has disgraced me” (P 14).*

When the sexual relations between the partners is disrupted because one of them has a sexual defect, they may begin accusing each other of infidelity and sometimes physically harming the children. The chronic adverse condition may lead to frustration and the feeling of being worthlessness and may lead to suicide attempts.

*“My husband has intercourse with me multiple times every day and even many times during the night. He doesn’t let me sleep. He doesn’t understand me, and when I don’t let him do it he accuses me of having sex with someone else and even beats me. I have had an unsuccessful marriage and my daughter from my first marriage lives with us. When I don’t let him have sex with me, he uses it as an excuse to beat my daughter. I tell him he is sick and needs to see a doctor, but he doesn’t accept he has a problem.” (P 17).*

Another participant said:

*“After two years of living with my wife, I realized she was cheating. I told my family two months ago that I did not want to live with her because she was cheating. I caught her texting two nights ago, and she said I was mistaken and it was the other guy that was bothering her. I beat her. I broke her arm, and she is in hospital now. I cannot tolerate her anymore because she cheats all the time. Yesterday, my mother insisted that I go visit her, but I said no. My family thinks it is my fault. I tell them she is cheating, but they do not believe me. I went home and poisoned myself with paraquat. I don’t have a job, and my family supports me. Instagram and Telegram have become a real problem for me.” (P 15).*

#### Failure in romantic relationships

Based on the interviews, failure in romantic relationships occurs when two people have an intimate relationship, which may sometimes be accompanied by sexual relationships as well and one of them realizes that the other has relationships with someone else. This leads to conflicts, arguments, quarrels, and suicide attempts. When their intention is marriage, their relatives know about the relationship, so when the marriage is called off, the individual considers it a disgrace, especially when they think they have lost a good marriage opportunity and sometimes, the betrayed person finds out that their partner has a relationship with someone with a higher social status, feels humiliated and worthless, and attempts suicide.

*My boyfriend cheated on me; we had been together for ten years and were supposed to get married. Now, he is with a widow who even has children. He has had sex with her. He always swore that he only loved me and that he was only in a relationship with me. Everyone in the village knows that we were seeing each other, and my family knows that we were friends; I have missed so many opportunities for him. Now, my family keeps telling me ‘We told you not to do this.’ I called him, but that woman answered the phone. I cannot believe it.” (P 2).*

Another participant stated:

*“I met a boy in the company five years ago, and he proposed this year. Yesterday, he called me, and I told him I was with my nephew. He called ten minutes later and claimed there was someone else there with me too. I swore there was no one, but he called off the engagement. We had told everyone there would be an engagement ceremony, and I was humiliated. Our relatives call and ask ‘What happened? Didn’t Y get engaged?’ I love him so much.” (P16).*

### Emotional trauma

Another sub-category of instability in romantic relationships is emotional trauma. This means that an individual has lost a friend or relative to whom they had a considerable emotional attachment. Two examples are not receiving support from the family and facing adversity when familial support is expected in hardships. Another is being constantly sexually harassed and threatened. These conditions are not tolerable and eventually lead to suicide attempts.

#### Lack of familial support (rejection by the family)

Where families are expected to play a protective role for their children, some experience insult, humiliation and blame by family members and others. Seeing others (friends and acquaintances) receiving familial support, feeling helpless to change the situation could result in feelings of disgrace, worthlessness and insignificance, thus potentially resulting in a suicide attempt. The person may argue with their family when they do not have the family’s support and might even be punished or insulted for their problems. If the situation does not change, experiencing further conflict and humiliation could increase the desperation and even lead to increasing intensity feelings of disgrace, worthlessness, and insignificance, and eventually lead to suicide attempt.

*“I have problems with my father; these problems are related to money, treatment, and work. I go to university, and it is far; he does not give me any pocket money and does not say ‘Take this money and buy something to eat if you get hungry on the way.’ What kind of father is that? He compares me with other people’s daughters. He says, ‘They have gotten married, but look at your problems.’ I cannot take this anymore. The new semester began, and my mother said, ‘Where the hell do you go every day?’ even though I always came straight home because I am afraid I would run out of money or someone would kidnap me or catcall me. When I go to the university, my father says to our relatives ‘God knows where she is going!’” (P 3).*

#### Death of relatives

The death of parents, death of relatives to whom a person is emotionally attached, the death of one parent resulting in the remarriage of the other, forcing the children to live with relatives who will reprimand them for what were ordinary behaviours in their own home, or the parent’s unwillingness to visit their children even after remarriage can cause feelings of worthlessness, burdensomeness, and insignificance, which may lead to *attempting suicide.*

*“My mother died About 5 or 6 years ago, I used to live with my stepmother, who didn’t treat me well. Since then, I’ve been living with my grandparents. My grandfather passed away 6 months ago, and I’ve been really upset during these few months because my grandfather wasn’t there anymore and my father didn’t answer my messages. My brother is married and lives in another city. He says that I shouldn’t count dad as family anymore, but I can’t. I’ve been calling and texting my dad for two months, but he hasn’t answered yet. I’ve been thinking about suicide for the past one or two months. My grandfather isn’t here with me anymore and my father doesn’t care about me. It doesn’t matter if exist or not.” (p 18).*

*“One of my sisters is married, and the other is divorced and lives with my other sister. I am homeless and tired of staying in different places. I wish I were dead. I have been living with my grandmother for a while now; they have lived a different life. When you are not allowed to take anything from the refrigerator, you cannot call that place your home. I am not allowed to mention my mother’s name. My grandmother criticizes my hair and asks me, ‘Why are your pants short?’ They have opinions about everything. My mother died one year ago, and my father several years before that. My mother provided for several families; she was a fortune teller. If she were alive, we would not be living in these circumstances.” (P 5).*

#### Sexual assault

People expect to be supported by their relatives, but sometimes they are abused by them. Sometimes, someone who has committed a crime and is in prison expects to be safe in prison, but they are sexually assaulted there. This can create a feeling of worthlessness, leading to suicide attempts.

Another is being constantly sexually harassed and threatened. These conditions are not tolerable that can lead to feeling of worthlessness and eventually lead to suicide attempt.

*“My uncle has been harassing me for 7 or 8 years; he touches me when my parents are away and says, ‘You are mine.’ He even has the key to our house and comes in when I am alone, especially recently when my father was hospitalized. He threatens to hurt my family if I tell anyone. I am really tired; I hate myself. I cannot fight him back, so I decided to kill myself.” (P 6).*

Another participant had been sexually assaulted in childhood and later assaulted others; he felt guilty and worthless:

*“Yesterday, my commander tore my transfer letter without any reason and told me to get lost. I attempted suicide. I am tired of living. Since my military service started, I have been feeling guilty for what I have done. I have had sex with children against their will. I may have done it a hundred times. It started two years ago. When I was a child, others in my neighborhood did the same to me, so I did it to others when I grew up. I went to prison for assault; it was a youth detention center; they raped me even there.” (p 7).*

### Loss of emotional resilience (emotional failure + emotional trauma)

Another sub-category of instability in emotional relationships is loss of emotional resilience, which occurs when someone experiences both emotional trauma and emotional failure in a short period of time; this may lead to repeated self-injury and suicide attempts, and life loses its meaning for the person.

Loss of emotional resilience, the ability to adapt to stressful situations, is the third subcategory of instability in emotional relationships. This occurs when the person experiences blame, insult, humiliation, betrayal, and disloyalty over time from different people who are expected to be supportive, respectful, and loyal to them. As a result, they repeatedly lose their sense of belonging, see themselves as burdens to others, and feel that nobody cares about them, which can eventually lead to attempting suicide.

#### Vicious cycle of problematic emotional events (death of friends or relatives, rejection by family, and failure in romantic relationships at the same time)

When someone has both lost a family member or an intimate friend and experienced failure in a romantic relationship within the past few months, there is a higher chance that they may attempt suicide.

One participant had experienced betrayal in an emotional relationship, and his partner had married someone with a better social status; he was continuously reproached by his family, experienced great emotional pressure, and felt he was worthless to his family and the person he loved; as a result, he attempted suicide:

*“My sister passed away several months ago; I loved her a lot; then, someone I loved got married. I liked her, but she told me, ‘You do not have anything of yourself.’ I hated those words; they burned me to the core. After that, I did not like her anymore. At home, everyone told me, ‘You have not done anything to prepare for the university entrance exam.’ They scolded me all the time. I was looking for an excuse, and I wanted to kill myself; I even injured myself several times.” (P 8).*

When someone loses an intimate friend, which affects their resilience in dealing with problems, and they have familial issues too, they may repeatedly attempt self-injury and suicide. Moreover, when due to parents’ divorce, someone lives with relatives who have a supporting role in their life and they are emotionally attached to, they may attempt suicide if these relatives die.

*“I have all kinds of problems. I had a fight with my father six months ago. I wanted to kill myself so many times. Then my friend killed himself; we were very close, and after my friend died, I started injuring myself. A couple of days later, my family took me to an addiction treatment camp and said I was an addict, but I wasn’t. The girl I loved is married now and has a daughter; I met several girls after her, but they could not fill her place. My family does not understand me, and my friend is gone. What kind of life is this? I am always in detention centers, camps, or hospitals. My life does not mean anything anymore. I’d rather not live anymore.” (P 9).*

#### Familial crisis resulting from divorce

When someone has problems with their spouse, they are not happy with their life, they cannot do anything to change the circumstances, and their family treats them differently after the divorce, they will face many conflicts, disputes, and limitations.

*“I have gotten divorced and have not seen my children for 18 days. My husband used to beat me. We were married for two years and had problems from day one. My family used to limit me before my marriage, and it has gotten even worse now; I hate my family. I haven’t been able to go anywhere alone; I always go out with my mother. My brother believes that everything is different for me now; he says, ‘A divorced woman is not like she used to be, and people might talk behind her back,” so they limit me even more now. My life was awful with my husband. He did not give me money; he did not take me out shopping; I was allowed to go only to my mother’s and return whenever he told me to. I lived with my husband’s family. I have attempted suicide several times.” (P 10).*

Another participant, who had gotten divorced twice and was continually reproached by her family, felt that she was unwanted, worthless and insignificant.

*“No one wants me, not even my daughter. I was forced into marriage at 11, and my husband did not treat me well. He did not buy me anything. When I got divorced, my child said she did not want to live with me. My family kept blaming me. They quarreled with me so much that I married our neighbor’s son to get away. He was an addict. He used to hallucinate and beat me every day. I got divorced again. My mother got worse and took to sarcasm. They said I was an extra mouth to feed. Now my father does not talk to me. My sisters, who are married, reproach me when they come to see us. They tell me not to go out, because people will talk behind a widow’s back. I gave my divorce money to someone to invest for me, but he has gone bankrupt and does not give my money back. Now, I cannot even go out with my friends because I do not have any money. I have attempted suicide several times. I will do it again when I get well. I do not have anywhere to live. If I did, I would not live in that house. I am unwanted and a burden.” (P 4).*

#### Disruption in emotional relationships (failure in a romantic relationship along with a lack of support by family)

When someone experiences failure and betrayal in a romantic relationship and has experienced their parents’ divorce in recent months or their parents are addicts and they experience conflicts and disputes at home, they feel that they do not matter, and there is a higher chance that they may attempt suicide.

One participant had experienced failure in a romantic relationship, and his father was an addict; he did not have his family’s support. He was even responsible for providing for the family.

*“I was thinking about it for almost a month until yesterday when no one was home, I took the pills. I do not like to live in this world anymore. I am a laborer and my father is an addict. I was going to get engaged, but it did not work out. My father does not care; he is an addict and has stained my reputation. I provide for the family; we live in a village. Why should I stay and go through so many hardships when no one cares? There is no hope. I have worked in different cities and in different jobs since I was 12, but nothing good came out of it. What good is this life? My father is irresponsible; there is no uncle, mother, or anyone else; I have not been a bad person or done anything wrong.” (P 11).*

Another participant had experienced failure in a romantic relationship and felt that his father did not understand him; he was limited in socializing with friends and had many disputes with his father.

*“I am tired of living because of my father and my family’s criticisms. My father treats me like a 5-year-old; I am not allowed to go out and have friends or do the things I want. I want to marry a friend from university. We are Sunni, and since she is Shia, my father is against it. Now, even the girl does not want to marry me. She gave up on me a month ago. I have to fight with my father if I want to go out with my friends.” (P 12).*

#### Familial crisis resulting from disability

When someone is physically disabled and depends on their family for their basic needs, but their family does not support them; the pressure that they experience may drive them towards attempting suicide.

*“I fell from a tree and suffered a spinal cord injury. I could not work anymore. My wife is cheating on me, and she wants a divorce now. I am tired of living. My life was not like this before. Once, I used to help others, and now I need others’ help. I do not want to live anymore; I do not matter to anyone.” (P 13).*

## Discussion

The present study results indicated that instability in emotional relationships had led to self-poisoning suicide in young people, and this concept was extracted based on the participants’ experiences and perceptions. Instability in emotional relationships had a central role in attempted self-poisoning. It is usually associated with feelings of humiliation, worthlessness, disgrace, and the feeling that one does not matter. A change in communication distance (reduction in closeness or intimacy) can predict the nature and severity of consequences of suicide [10].

Betrayal in couples’ relationships can lead to conflicts, quarrels, and eventually suicide attempts. Sometimes, one of the spouses accuses the other of betrayal, which results in impulsive suicide in the accused party. Impulsive suicide may happen when one of the spouses accuses the other of infidelity in the presence of others, making them feel debased and defamed and possibly driving them towards an impulsive act of suicide at a time close to the accusation being made. Sometimes, a person might impose limitations on their spouse’s social relationships to prevent betrayal, leading to conflict, quarrels, and attempted suicide. In such situations, people usually feel worthless. In one study, three-quarters of the participants mentioned communication problems related to feelings, such as betrayal, rejection, shame, and the spouse’s failure in their responsibilities, as the main reason for attempting suicide. When couples have problems in their relationship (e.g., communication breakdown), these problems can increase the risk of suicide [27]. Relationship problems are one of the main causes of suicide [27, 28]. These studies have generally suggested that infidelity and problems in couples’ relationships can lead to suicide; however, the most important issue discussed in the present study is suicide due to feelings of disgrace and worthlessness. In some cases, the individual tried to change the conditions that had led to these feelings. The resulting conflict may cause more humiliation, and ultimately, being unable to change the conditions can lead to suicide. When individuals cheat on their spouse, they hide it. Being accused of betrayal can cause feelings of disgrace and worthlessness in some cases and lead to suicide attempts because it can change how people treat that person. If an individuals’ social interactions are limited by their spouses to prevent betrayal in a way that their relatives realize it, conflicts can break out between the couples over the limitations. If these limitations are not removed, others might assume that the individual is not trusted by their spouse, which leads to the feeling of worthlessness and, in some cases, suicide attempts.

Failure in romantic relationships resulting from betrayal or break-up by one party creates stressful conditions for the other and increases the chances of suicide attempts. In one study, people who had experienced failure in romantic relationships in the previous 3 months had a high risk of attempting suicide due to depression [29]. People who are in romantic relationships usually hide this relationship from their family, relatives, acquaintances, and people around them. In some cases, if others find out about the romantic relationship and they break up, it can have consequences, such as being blamed for the break-up, for the individual; moreover, their families might limit their social interactions, leading to the loss of many marriage opportunities. Furthermore, others may assume that these individuals will be unfaithful to their spouses in the future.

Those who attempt suicide in front of a spouse or lover are often reacting to some reduction in closeness – what Black calls “under-intimacy.” Under-intimacy includes separation, divorce, and infidelity, all of which cause severe conflict between intimate partners [10, 11]. The abovementioned study only pointed to severe forms of conflict, whereas in those with a deep emotional relationships, encountering interpersonal factors cause them to feel worthless, disgraced, and unimportant to others as well. This leads to an effort to change the situation and can even be accompanied by conflict and physical argument. Fruitless efforts and the feeling of disappointment can lead to suicide attempts in individuals.

A lack of support by the family, being rejected by the family, or divorce and addiction problems in the family, when accompanied by a lack of emotional support for children, put a lot of stress on individuals; these problems are usually associated with feelings of not being understood, being worthless, and being a burden, which can increase chances of attempting suicide. One factor related to suicide in youth is the loss of parents or their divorce [30]. Young people who believe that their parents do not care for them or control them too much (affectionless control) have more mental distress and are at a higher risk of suicidal thoughts and behaviors than their peers [14]. Previous studies have reported a significant relationship between increased suicide risks in adolescents and parental factors such as low income and familial support [31]. Familial unity and integrity and familial emotional support are protective factors against suicide and depression. Enhancing unity and integrity in families can efficiently prevent suicidal thoughts, depression, and high-risk behaviors [32]. In Iran, parents must be over 60, or the father has to have passed away for children to be financially supported by government organizations. However, there is no governmental support for families with addicted or homeless fathers. In such cases, children are in undesirable conditions, must provide for their families, and have to experience educational and social problems, such as addiction.

When someone experiences emotional failure and emotional trauma within a period of several months, and their emotional resilience is affected, their lives become meaningless, and they may repeatedly injure themselves and attempt suicide. People who experience significant stressors are at higher risk of attempting suicide [31]. A study reported that people who experience constant communication problems experience more suicide attempts and longer suicidal periods [27]. The present study indicated that suicide attempts may result when the individual experiences various forms of emotional stress caused by the people who are expected to be loyal, respectful, and supportive to them but show infidelity, betrayal, insult, humiliation, and lack of support because the individual will experience feelings of disgrace, worthlessness, insignificance, and humiliation. Moreover, experiencing these feelings more frequently can lead to repeated suicide attempts and even mental problems. In another study, having meaning in life was associated with decreased emotional stress and suicide risk [33]. In this study, when people experience several instances of emotional stress, life does not mean anything to them, and they feel that they are worthless, they are a burden, they have been humiliated, and they do not matter to others.

This study revealed that the loss of parents and intimate friends to whom people are emotionally attached leads to emotional trauma; moreover, when someone experiences the death of their parents and is not supported by their relatives, they lose their emotional resilience; this is associated with a feeling of being a burden and eventually leads to suicide attempts. Children who lose their parents and receive little support, those who are only children or single, and people who experience frequent changes in their marital status are at high risk of attempting suicide compared with people who have high levels of social support [34]. In one study, it was reported that although the majority of people who experience their parents’ death return to normal life after a period of grief, the risk of attempting suicide increases in them [33–37]. The loss of integrity in families of people who have lost a relative also increases the risk of suicide [34].

Based on the interviews, there is an expectation of support from relatives. However, some are abused by the relatives instead. The same can happen for a person who expects to feel safe while in prison yet experiences sexual abuse instead. These situations can create a feeling of worthlessness, leading to suicide attempts. The youth who feel that they have close relationships with their peers, parents, and family members and youths who feel they are part of their school or society are less likely to have serious thoughts about suicide than their counterparts [14, 15].

According to one participant who had a disability, disabilities can lead to dysfunction, economic problems, divorce, spouse’s infidelity, and rejection or blame by the family, and are associated with feelings of being insignificant, lonely, unwanted, and worthlessness and a burden to others, which may lead to the decision to attempt suicide. Kazhem’s (2017) work suggests that the relationship between physical disability and suicide is beginning to be examined within empirically supported frameworks of suicide and reports that interpersonal factors (e.g. perceived burdensomeness) and pain are mechanisms contributing to this heightened risk of suicide [38].

The notable point in this study is that instability in emotional relationships creates the feeling that a person does not matter to the family, and the feelings of disgrace, humiliation, worthlessness, and burdensomeness may lead to suicide attempts. The interpersonal-psychological theory of suicidal behavior introduced by Joiner posits that people tend to attempt suicide due to feelings of burdensomeness (being a burden to others) and thwarted belongingness (not belonging to a social group) [37]. The results of this study showed that a thwarted sense belonging and the feeling of being a burden to others lead to suicide attempts because they are accompanied by the feelings of disgrace, worthlessness, and insignificance. The more emotional stress one is subjected to, the less sense of belonging they feel and the more serious suicide attempts they will have. In Joiner’s theory the feeling of belonging is so powerful that, when satisfied, it can prevent suicide even when perceived burdensomeness and the ability to enact lethal self-injury are in place [12]. Based on the result of this study, burdensomeness is felt when one has lost all of their belongings and is usually accompanied by feelings of being unwanted, valueless, unimportant, lonely, and a burden to others, often leading to repeated serious suicide attempts.

Fleischer (2000) focuses on the act of suicide as a normal way of communication in an extreme situation when the persons in question see verbal expression as insufficient [39]. In this study, an extreme situation is one in which an individual expects respect and support from other people who are in this situation themselves, but instead, they are blamed, betrayed, raped, rejected, and deceived. Consequently, the individual experiences feelings of worthlessness, disgrace, humiliation, and insignificance and as a result, might attempt suicide.

In general, being accused of betrayal by a spouse or being labeled in the community (family, relatives, and acquaintances) as a failure in romantic relationships can negatively affect an individual’s social position, value, and respect and lead to changes in the behavior of people or blame by others. Therefore, the individual feels disgraced and worthless (lack of or decreased social value), which can lead to suicide attempts. Lack of familial support and being blamed and rejected by family members exacerbate the negative feelings, can make the individual feel that they are a burden, that they do not belong, or that they are not important to others, and eventually can result in frequent and serious suicide attempts. Individuals expect to be supported and respected by their family and spouse in the community.

Different emotional stressors have been mentioned in different studies as factors causing suicide, but how these stressors lead to suicide and the feelings experienced by these people resulting in their suicide have not been discussed. However, the present study revealed that when individuals experience one or more interpersonal emotional stressors, they attempt suicide because these stressors lead to feelings of disgrace, worthlessness, insignificance, burdensomeness, and humiliation caused by the people who are expected to be loyal, respectful, and supportive. This highlights the need for more studies in this field.

### Limitations

This study is the first of its kind in the west of Iran; however, it is limited by the small sample size and the challenge of interviewing some patients because they were discharged with personal consent. The qualitative nature of the study limits its generalizability to the broader population. However, efforts were made to enhance the representation of different voices and concerns of participants in order to increase the credibility of the data. Moreover, by clearly stating these study limitations, data collection and analysis methods, participant selection, and subject description, we hope transferability would be possible so other researchers may continue further work in this area.

## Conclusion

The present study results provided a deep understanding of the role of instability in romantic and familial relationships in suicide attempts. Some information on risk factors and warning signs of interpersonal stressors is provided for psychiatrists and nurses dealing with suicidal patients. The findings of this study can provide a context for quantitative and qualitative studies on education on and prevention of suicide. Social support can effectively reduce and moderate emotional stress, and the media, medical teams, and schools can be effective in preventing suicide by educating proper communication skills among family members and those with intimate relationships. Moreover, families with addiction problems must receive social support.

## Data Availability

The datasets generated and/or analyzed during the current study are not publicly available due to the University of Social Welfare and Rehabilitation Sciences’ policies, but are available from the corresponding author on reasonable request.

## Abbreviation

WHO: World Health Organization
